# Relationship between iron deficiency and severity of tuberculosis: Influence on T cell subsets

**DOI:** 10.1016/j.isci.2024.111709

**Published:** 2024-12-30

**Authors:** Zheyue Wang, Zhenpeng Guo, Qiang Zhang, Chenchen Yang, Xinling Shi, Qin Wen, Yuan Xue, Zhixin Zhang, Jianming Wang

**Affiliations:** 1 Department of Epidemiology, Key Laboratory of Public Health Safety and Emergency Prevention and Control Technology of Higher Education Institutions in Jiangsu Province, Center for Global Health, School of Public Health, Nanjing Medical University, Nanjing 211166, China; 2 Changzhou Medical Center, Nanjing Medical University, Changzhou 213004, China; 3 Department of Pulmonary Diseases, The Third People’s Hospital of Changzhou, Changzhou 213001, China; 4 Department of Epidemiology, Gusu School, Nanjing Medical University, Nanjing 211166, China; 5 National Vaccine Innovation Platform, Nanjing Medical University, Nanjing 211166, China

**Keywords:** Immunology, Microbiology

## Abstract

Tuberculosis (TB) remains a leading cause of death globally, with nearly half of TB patients experiencing iron deficiency. The role of iron supplementation as an adjunct therapy remains controversial. This study examines the impact of iron deficiency on TB progression and immune function. We conducted a case-control study involving 808 pulmonary TB patients recruited from Changzhou Third People’s Hospital (2018–2022) to investigate the association between serum iron levels and TB severity. Additionally, we evaluated the relationship between baseline serum iron levels and pulmonary lesion characteristics during antituberculosis treatment using a cohort study of 89 patients. We observed that low serum iron was associated with more severe lung symptoms, decreased MAIT, Vδ2+, and Treg cell percentages, and increased interleukin-1β (IL-1β) and IL-7 levels. Findings suggest that iron deficiency may exacerbate lung lesions by altering T cell subsets and cytokine profiles.

## Highlights

• Iron-deficient TB patients present with more severe clinical symptoms and chest CT imaging
• Iron-deficient TB patients exhibit a significant reduction in MAIT, Vδ2^+^ γδ T cells, and Treg
• The immunophenotype of MAIT is different in TB patients with or without iron deficiency

## Graphical abstract

**Figure:**
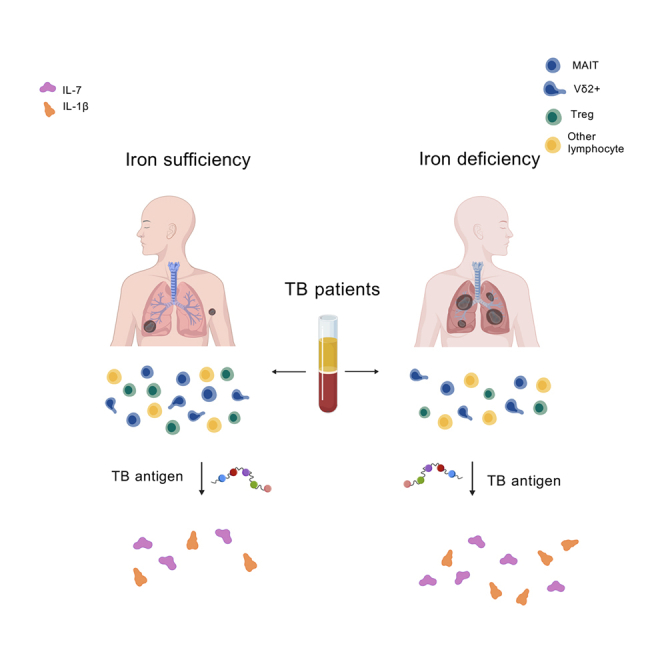

## Introduction

According to the World Health Organization’s tuberculosis (TB) report, approximately 10.8 million individuals developed active TB in 2023, resulting in 1.3 million deaths globally. The disease burden was disproportionately high, with 87% of cases concentrated in just 30 countries, including China, which accounted for 7.1% of these cases.^1^ Although the causes of TB are complex, trace elements, including iron, play a vital role in the occurrence and development of the disease. It is worth noting that about half of TB patients suffer from iron deficiency.^2^^,^^3^^,^^4^^,^^5^

Iron is indispensable for both human physiology and the survival of *Mycobacterium tuberculosis* (*M.tb*), which utilizes siderophores to scavenge iron from host cells.^6^ Additionally, *M.tb* can acquire iron through the phagocytosis of aged red blood cells by macrophages, known as erythrophagocytosis.^7^ Previous research has primarily focused on iron uptake mechanisms of *M.tb* and the associated redox disorders linked to ferroptosis,^8^ but the role of iron deficiency in TB pathogenesis remains underexplored and inconsistent across epidemiological studies.^9^^,^^10^^,^^11^

Iron is vital for numerous physiological processes, including erythropoiesis, hemoglobin synthesis, and immune function. It promotes the polarization of macrophages to the M1 phenotype,^12^ facilitates natural killer (NK) cell activation,^13^ and accelerates the proliferation of T and B cells.^14^ Upon infection, innate immune cells, particularly macrophages, recognize bacterial components through pattern recognition receptors (PRRs), forming phagosomes where *M.tb* can survive. Activated T cell responses, particularly those involving CD4 T cells, play a pivotal role in determining the outcome of *M.tb* infection.^15^ Other T cells and cytokines, such as IFN-γ and IL-1, are also crucial in TB pathogenesis,^16^ suggesting that iron likely regulates these immune responses and thus influences TB progression.

This study evaluates the impact of iron deficiency on the severity and prognosis of TB in a Chinese population. It explores its influence on T cell immunity and cytokine profiles, aiming to provide evidence for the potential use of iron supplementation in antituberculosis therapy.

## Results

### Poor nutritional status of patients with severe TB

We enrolled 808 active pulmonary TB patients, comprising 666 mild and 142 severe cases. Both groups were initially evaluated using a standard medical checklist before antituberculosis treatment (Table S1). Of the participants, 580 (71.78%) were males and 228 (28.22%) were females, with no significant differences in sex and age distribution. Analysis of liver, renal, and cardiac functions revealed differences between the groups, specifically in adenosine deaminase (ADA), glutamyltransferase (GGT), and lactate dehydrogenase (LDH) levels. Further examination of indicators beyond normal ranges indicated that TB patients exhibited a poor nutritional status, with deficient levels of iron, transferrin, albumin, and prealbumin, which were even lower in the severe condition group. The mild condition group also had higher apolipoprotein A and platelet distribution width (PDW). Notably, C-reactive protein (CRP) levels were ten times higher in the severe group, indicating a heightened immune response (Table 1).

**Table 1:** Characteristics of mild and severe TB

Variables	Mild TB (*n* = 666)	Severe TB (*n* = 142)	Normal reference range	*p* value
**Demography**				

sex	–	–	–	0.078
male	469 (70.4%)	111 (78.2%)	–	–
female	197 (29.6%)	31 (21.8%)	–	–
age	53.5 [34.0, 68.0]	57.0 [32.5, 73.0]	–	0.148

**Nutritional status**				

glucose (mmol/L)	4.90 [4.40, 5.60]	5.00 [4.52, 5.76]	[3.89, 6.11]	0.144
total protein (g/L)	66.7 [63.2, 70.6]	65.8 [60.5, 69.8]	[65, 85]	0.030
albumin (g/L)	37.4 [33.6, 40.3]	31.4 [27.8, 34.7]	[40, 55]	<0.001
globulin (g/L)	29.6 [26.1, 32.8]	33.7 [29.6, 38.5]	[20, 40]	<0.001
A/G	1.3 [1.0, 1.5]	0.9 [0.8, 1.1]	[1.2, 2.4]	<0.001
prealbumin (mg/dL)	19.6 [14.9, 23.9]	12.1 [8.9, 17.5]	[20, 40]	<0.001
phosphorus (mmol/L)	1.12 [0.97, 1.24]	1.03 [0.92, 1.16]	[0.8, 1.45]	<0.001
calcium (mmol/L)	2.17 [2.10, 2.25]	2.09 [2.01, 2.20]	[2.0, 2.5]	<0.001
potassium (mmol/L)	3.91 [3.69, 4.15]	3.90 [3.63, 4.22]	[3.5, 5.3]	0.818
sodium (mmol/L)	141 [139, 142]	138 [135, 141]	[137, 147]	<0.001
chlorine (mmol/L)	105 [103, 107]	102 [98, 104]	[99, 110]	<0.001
magnesium (mmol/L)	0.88 [0.81, 0.93]	0.84 [0.78, 0.90]	[0.62, 1.05]	<0.001
iron (μmol/L)	10.20 [6.58;15.10]	5.04 [3.58, 8.05]	female [9, 27]; male [11, 30]	<0.001
transferrin (g/L)	2.00 [1.70, 2.20]	1.60 [1.30, 1.87]	[2, 4]	<0.001
total cholesterol (mmol/L)	3.86 [3.42, 4.50]	3.44 [3.02, 4.00]	[3.4, 5.8]	<0.001
triglyceride (mmol/L)	1.04 [0.75, 1.39]	0.96 [0.80, 1.25]	[0.56, 1.7]	0.368
HDL (mmol/L)	1.04 [0.86, 1.25]	0.82 [0.69, 1.03]	[0.78, 2]	<0.001
LDL (mmol/L)	2.29 [1.92, 2.83]	2.01 [1.70, 2.50]	[0, 3.7]	0.002
apolipoprotein A (g/L)	0.87 [0.76, 1.01]	0.68 [0.61, 0.82]	[1, 1.6]	<0.001
apolipoprotein B (g/L)	0.70 [0.61, 0.84]	0.69 [0.62, 0.80]	[0.6, 1.2]	0.858
lipoprotein a (mg/L)	141 [70.5, 298]	208 [96.5, 470]	[0, 300]	0.002
free fatty acid (mmol/L)	0.37 [0.25, 0.48]	0.44 [0.28, 0.54]	[0.1, 0.77]	0.014

**Blood cells and immunity**				

PDW (%)	11.9 [10.5, 13.4]	10.7 [9.53, 11.9]	[14.8, 17.2]	<0.001
CRP (mg/L)	5.3 [0.9, 26.7]	51.1 [26.8, 81.8]	[0, 5]	<0.001
leukocyte (10^9^/L)	5.9 [5.0, 7.3]	6.5 [5.2, 8.3]	[4.1, 11]	0.006
lymphocyte (10^9^/L)	1.5 [1.1, 1.9]	1.0 [0.7, 1.3]	[1.2, 3.8]	<0.001
neutrophil (10^9^/L)	3.7 [2.8, 4.8]	4.7 [3.5, 6.0]	[1.8, 8.3]	<0.001
monocyte (10^9^/L)	0.5 [0.4, 0.7]	0.7 [0.5, 0.8]	[0.14, 0.74]	<0.001
eosinophils (10^9^/L)	0.11 [0.07, 0.19]	0.07 [0.02, 0.13]	[0, 068]	<0.001
basophil (10^9^/L)	0.03 [0.02, 0.04]	0.02 [0.02, 0.03]	[0, 0.07]	0.002
IgM (g/L)	0.97 [0.70, 1.28]	0.85 [0.64, 1.04]	[0.29, 3.44]	0.205
IgA (g/L)	2.32 [1.80, 3.07]	3.08 [2.16, 3.68]	[0.72, 4.29]	0.004
IgG (g/L)	13.4 [11.3, 15.4]	14.1 [11.1, 16.0]	[8, 17]	0.362
complement 3 (g/L)	0.91 [0.76, 1.08]	0.96 [0.80, 1.15]	[0.79, 1.52]	0.331
complement 4 (g/L)	0.24 [0.20, 0.29]	0.27 [0.22, 0.34]	[0.16, 0.38]	0.098

The differences between mild and severe TB were analyzed using the Wilcoxon test for continuous variables and Fisher’s exact test for dichotomous variables. Data were shown as mean [interquartile range].

HDL, high-density lipoprotein; LDL, low-density lipoprotein; PDW, platelet distribution width; CRP, C-reactive protein.

**Figure 1:**
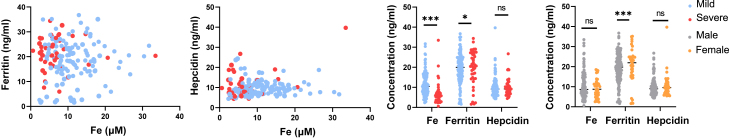
Relationships between serum iron, ferritin, and hepcidin in TB patients A total of 160 TB patients were enrolled (117 mild and 43 severe). No significant correlations were found between serum iron and ferritin (*p* = 0.27) or hepcidin (*p* = 0.24). Serum iron was significantly lower in severely ill patients (*p* < 0.001), while ferritin was higher (*p* = 0.016). Hepcidin levels did not differ between mild and severe patients (*p* = 0.816). Sex analysis (male = 122, female = 38) showed no differences in serum iron (*p* = 0.472) or hepcidin (*p* = 0.923) levels, but males had higher ferritin levels (*p* < 0.001). Differences were assessed using the Wilcoxon test. For iron, the unit is μM. Data were represented as individual values with a median (for the rightmost two figures). ∗, *p* < 0.05, ∗∗∗, *p* < 0.001, ns, not significant.

Since nearly half of the patients exhibited iron deficiency, this study mainly focused on serum iron levels. Serum iron regulation is complex and involves multiple systems. Ferritin, the primary storage form of iron, can indicate body iron content but increases during inflammation. To assess iron levels, we measured serum ferritin and hepcidin in 160 TB patients (117 mild and 43 severe). Initial Spearman correlation analysis found no significant relationships between serum iron and ferritin (*p* = 0.27) or hepcidin (*p* = 0.24). Further analysis showed that patients with severe conditions have significantly reduced serum iron levels (*p* < 0.001) but elevated ferritin levels (*p* = 0.016). No significant changes were observed in hepcidin levels (*p* = 0.816). Additionally, males had higher ferritin levels (Figure 1).

Considering the reverse trend of serum iron and ferritin in severely ill patients, we adjusted serum iron levels for ferritin to balance its influence. A matched-pair analysis, considering sex, age, and ferritin, confirmed that iron levels remained lower in severe patients (Table 2). Logistic regression analysis by adjusting for sex, age, and ferritin showed that serum iron had a protective effect against the severity condition, with an odds ratio (OR) of 0.81 (95% confidence interval [CI]: 0.72, 0.89; *p* < 0.001). The effect of ferritin was insignificant (*p* = 0.06) after the adjustment, with an OR of 1.17 (95% CI: 1.00, 1.38). This indicates that despite higher levels of inflammation, marked by increased ferritin, severely ill patients were still in an iron-deficient state.

**Table 2:** Serum iron, ferritin, and hepcidin levels in TB patients: matched-pair analysis

Variables	Mild TB (*n* = 55)	Severe TB (*n* = 39)	*p* value
Sex	–	–	0.484^a^
Male	39 [70.9%]	31 [79.5%]	–
Female	16 [29.1%]	8 [20.5%]	–
Age	60.0 [46.5, 70]	60.0 [36.0, 72]	0.806^b^
Fe	10.50 [7.78, 15.00]	5.76 [3.39, 7.64]	<0.001^b^
Ferritin	7.28 [1.97]	7.24 [2.10]	0.926^c^
Hepcidin	3.07 [2.57, 3.95]	3.24 [2.31, 4.41]	0.942^b^

a The differences between mild and severe TB were estimated by the χ^2^ test.

b The differences between mild and severe TB were estimated by the Wilcoxon test.

c The differences between mild and severe TB were estimated by the t test.

### Serum iron is negatively associated with High-resolution CT (HRCT) score

**Figure 2:**
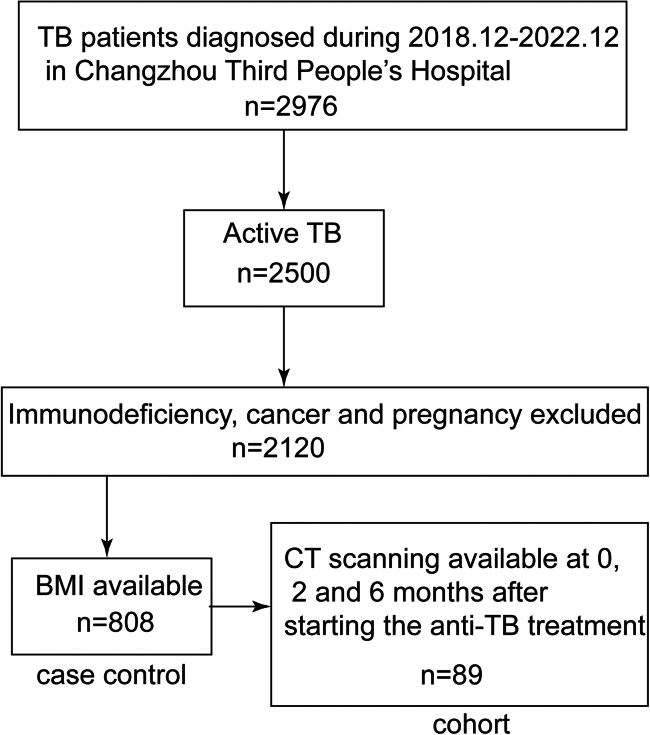
Patients involvement in the epidemiological study There were 2976 TB patients treated in Changzhou Third People’s Hospital from Dec 2018 to Dec 2022. We excluded non-active cases and those with immunodeficiency, cancer, or pregnancy. BMI was used in the modified Bandim TB score, and patients whose BMI was available were left for a case-control study. Only patients with CT scanning at 0, 2, and 6 months after starting the antituberculosis treatment were recruited in the cohort study.

To investigate the role of serum iron in TB progression, we conducted a cohort study to evaluate the changes in lung lesions using HRCT scores (Figure 2). Among the 89 patients (55 male, 34 female; median age: 45 years), we identified seven types of pulmonary lesions, including consolidation, micro nodule, nodule, ground-glass opacity (GGO), cavity, bronchiectasis, and parenchymal band. Micro nodules and consolidations were the most prevalent lesions, with median baseline HRCT scores of 3 and 2. The median overall baseline HRCT score was 9, which decreased to 7 at 2 months and 4 at 6 months, indicating a recovery process during antituberculosis treatment (Table S2). To better understand the relationship between disease recovery and serum iron levels, we used generalized estimating equations (GEE). We observed that the total HRCT score and the presence of consolidations and bronchiectasis were negatively associated with serum iron levels, suggesting that serum iron may be a protective factor during treatment (Table 3).

**Table 3:** Correlation between serum iron and HRCT scores

Variables	β (95% CI)	RR (95% CI)	*p* value^a^
Micro nodule	−0.06 (−0.14, 0.02)	0.94 (0.87, 1.02)	0.125
Nodule	0.01 (−0.07, 0.1)	1.01 (0.93, 1.10)	0.728
Consolidation	−0.18 (−0.27, −0.1)	0.83 (0.76, 0.90)	**<**0.001
Ground-glass opacity	0 (−0.01, 0.01)	1.00 (0.99, 1.01)	0.748
Cavity	−0.01 (−0.03, 0.01)	0.99 (0.97, 1.01)	0.256
Bronchiectasis	−0.07 (−0.1, −0.03)	0.93 (0.90, 0.97)	<0.001
Parenchymal band	0 (−0.02, 0.01)	1.00 (0.98, 1.01)	0.576
Total HRCT score	−0.31 (−0.53, −0.09)	0.73 (0.59, 0.91)	0.006

a The correlation was analyzed using generalized estimating equations (GEE). *p* values less than 0.05 were bolded to highlight significant results.

### Iron deficiency affects IL-1β and IL-7

**Figure 3:**
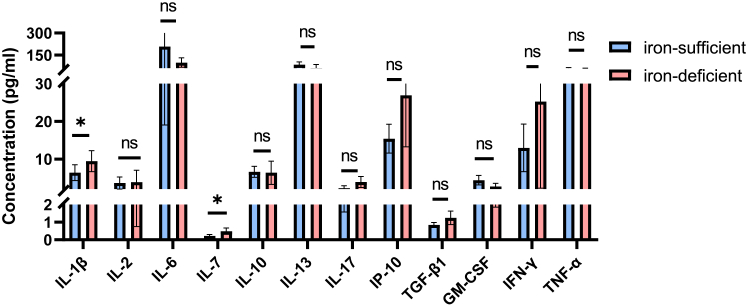
Cytokines produced by iron-deficient and sufficient patients PBMC of 48 TB patients (23 with iron deficiency, 25 with iron sufficiency) were isolated and stimulated with TB antigen CFP-10 and ESAT-6 peptides. Cytokines within culture supernate were measured using the BioLegend LEGENDplex kits and ELISA. Among these cytokines, IL-1β and IL-7 were much higher in iron deficiency TB (*p* = 0.029 for IL-1β and *p* = 0.014 for IL-7, Wilcoxon test). For TGF-β, the unit is ng/mL. Data were shown as mean with 95% CI. ∗, *p* < 0.05, ns, not significant.

According to the serum iron level, we divided patients into iron deficiency (*n* = 25, serum iron< 9 μM for women) and sufficiency (*n* = 23, serum iron ≥ 11 μM for men and ≥ 9 μM for women). As cytokines play essential roles in TB pathogenesis, we measured the levels of interleukin-1β (IL-1β), IL-2, IL-6, IL-7, IL-10, IL-13, IL-17, interferon gamma-induced protein 10 (IP-10), transforming growth factor-β1 (TGF-β1), granulocyte-macrophage colony-stimulating factor (GM-CSF), interferon γ (IFN-γ), and tumor necrosis factor-α (TNF-α) produced by peripheral blood mononuclear cells (PBMC) after antigen stimulation. Though IL-2 and IL-13 were twice as high in iron-sufficient patients, there was no statistical significance. IL-1β (4.94 pg/mL for iron sufficiency, 8.09 pg/mL for iron deficiency, *p* = 0.029), and IL-7 were higher in iron deficiency patients (0.19 pg/mL for iron sufficiency, 0.39 pg/mL for iron deficiency, *p* = 0.014) (Figure 3).

### Iron-deficient patients had less unconventional T cells

**Figure 4:**
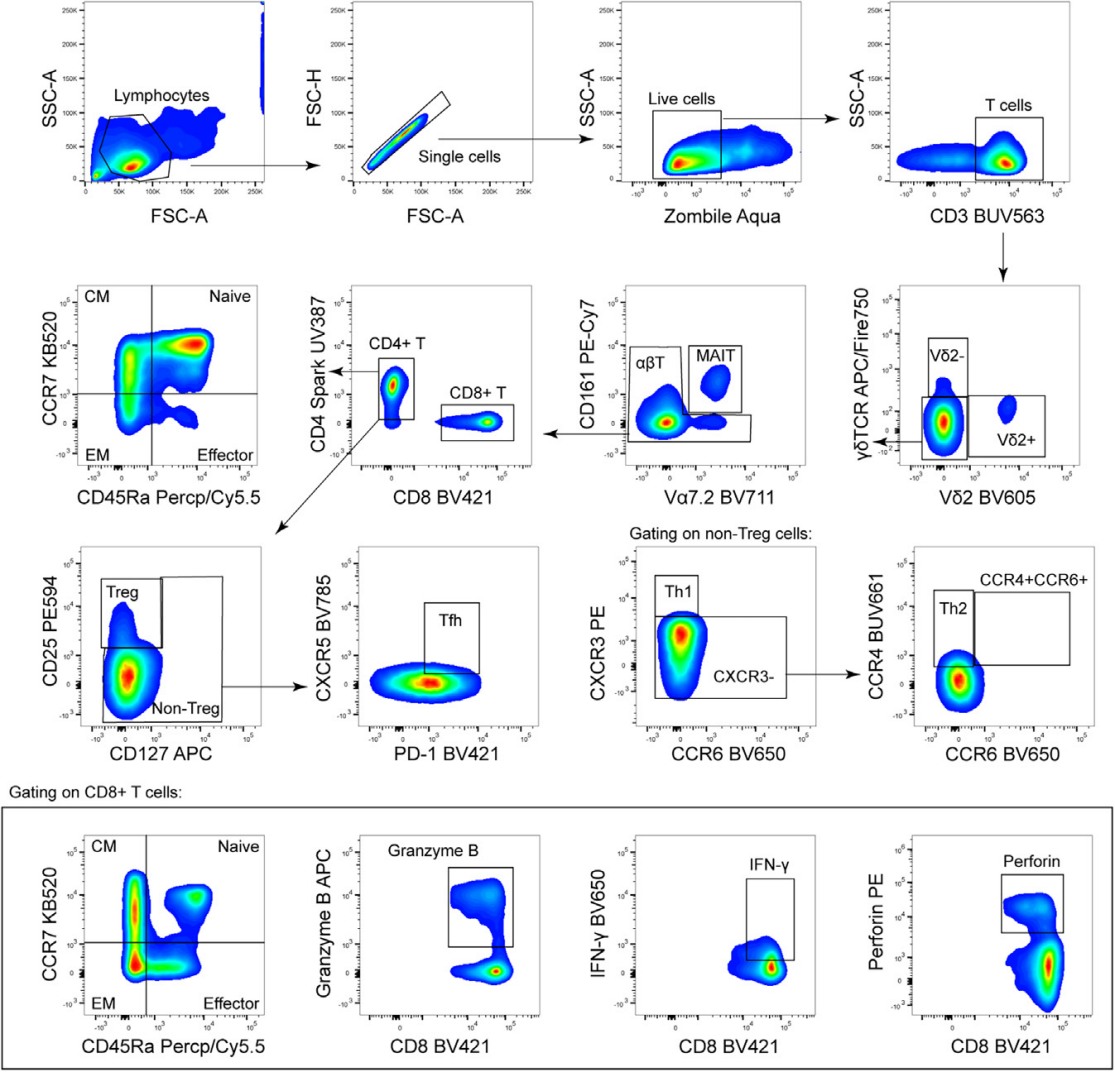
Gate of flow cytometry assay PBMCs from 48 patients were divided into two portions for differential staining: Panel 1 for CD4 T cell staining and Panel 2 for CD8 T cell staining. Lymphocytes were initially gated, and only single cells were included. Live cells were selected for subsequent T cell analysis. γδT cells were identified using γδTCR and Vδ2 markers. MAIT and αβT cells were separated from non-γδT cells based on Vα7.2 and CD161 expression. Classic CD4 and CD8 T cells were then gated from the αβT cell population. T cell differentiation into central memory (CM), effector memory (EM), naive, and effector subsets was determined by CD45RA and CCR7 expression for both CD4 and CD8 T cells. Regulatory T (Treg) cells were identified by CD25^+^ and CD127-expression, while non-Treg cells were further gated for T follicular helper (Tfh) cells by PD-1+ and CXCR5+ expression. Th1 cells were gated from non-Treg cells by CXCR3+, and Th2 cells were selected by CCR4+. The cytotoxic function of CD8 T cells was assessed by granzyme B, IFN-γ, and perforin expression.

Responses of T cells are predominant in host immunity against *M.tb*. We then evaluated the common T cell subsets CD4^+^ and CD8^+^ and their differentiation subsets—native, effector, central memory (CM), and effector memory (EM) T cells. Additionally, for CD4^+^ T cells, we analyzed Th1, Th2, and Tfh cells. The function of CD8^+^ T cells, namely, perforin, granzyme B, and IFN-γ expression, was also evaluated (Figure 4). There were no significant differences between iron-deficient and iron-sufficient patients regarding these conventional T cell subsets. However, unconventional T cells, such as mucosal-associated invariant T cells (MAIT) and γδT cells, exhibited different patterns. The proportion of MAIT cells in iron-deficient patients was 0.38%, significantly lower than the 0.67% observed in iron-sufficient patients. Vδ2^+^ γδT cells and regulatory T (Treg) cells also showed decreased frequencies in iron-deficient patients, suggesting that iron may exert a more significant influence on unconventional T cells after TB infection (Table 4).

**Table 4:** Distributions of T cell subsets between iron-sufficient and deficient patients

T cell subsets (%)	Iron-sufficient (*n* = 25)	Iron-deficient (*n* = 23)	*p* value
CD3^+^ IFN-γ	21.80 [10.60, 31.50]	20.30 [10.45, 26.65]	0.828
CD4^+^ T	67.70 [60.70, 72.60]	60.45 [55.57, 68.10]	0.133
CD4^+^ Naive	53.10 [42.40, 60.10]	54.70 [47.15, 63.95]	0.551
CD4^+^ CM	23.60 [18.50, 29.40]	25.80 [18.77, 31.18]	0.536
CD4^+^ EM	17.90 [15.80, 25.30]	17.50 [13.57, 20.78]	0.201
CD4^+^ Effector	1.67 [1.25, 2.91]	1.69 [1.00, 2.75]	0.749
Treg	4.72 [3.87, 5.95]	3.45 [2.98, 4.18]	0.016
Th1	0.46 [0.13, 0.55]	0.42 [0.18, 0.53]	0.991
Th2	10.40 [1.94, 14.30]	11.15 [3.55, 14.15]	0.941
Tfh	5.33 [2.55, 7.11]	5.24 [2.93, 6.45]	0.624
CD8^+^ T	32.70 [25.80, 38.10]	37.50 [26.25, 39.40]	0.322
CD8^+^ Naive	36.20 [20.50, 53.30]	29.70 [14.85, 55.05]	0.703
CD8^+^ CM	9.33 [5.05, 15.20]	8.94 [5.21, 14.30]	0.918
CD8^+^ EM	25.00 [20.00, 31.60]	27.00 [13.05, 36.10]	0.901
CD8^+^ Effector	18.80 [11.80, 33.90]	24.20 [13.75, 36.20]	0.529
CD8^+^ Perforin	37.40 [10.80, 76.60]	37.30 [21.80, 51.85]	0.869
CD8^+^ Granzyme B	35.50 [19.90, 59.30]	45.70 [32.00, 66.45]	0.219
CD8^+^ IFN-γ	50.80 [25.70, 59.70]	45.50 [28.75, 59.10]	0.926
MAIT	0.67 [0.39, 1.58]	0.38 [0.15, 0.54]	0.006
vδ2^+^	3.57 [2.00, 5.14]	1.63 [0.95, 2.75]	0.004
vδ2^-^	0.21 [0.10, 0.40]	0.36 [0.17, 0.52]	0.248

The differences between iron sufficiency and deficiency were estimated by the Wilcoxon test. Data were shown as mean [interquartile range].

### MAIT cells exhibited distinct dominant clusters in iron-deficient versus iron-sufficient patients

**Figure 5:**
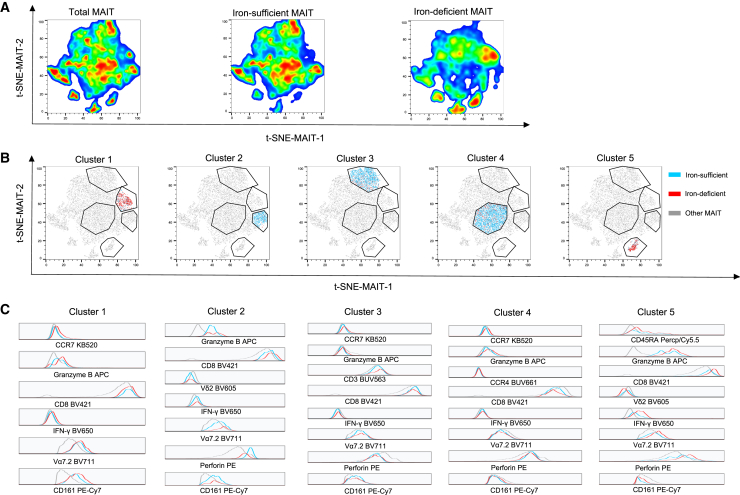
MAIT cells in iron-deficient and sufficient patients The number of MAIT cells was significantly reduced in iron-deficient TB patients. We concatenated all 25,610 MAIT cells from all patients to perform a t-SNE analysis. (A) Distinct clusters were observed between iron-deficient and sufficient patients. (B) Five distinct clusters were identified for a detailed study. Clusters 1 and 5 were more prevalent in iron-deficient patients, while clusters 2, 3, and 4 predominated in iron-sufficient patients. (C) The immunophenotypes of the five clusters in both patient groups were further analyzed. MAIT cells in clusters 1, 2, and 5 were more active, characterized by higher cytokine production, whereas clusters 3 and 4 were relatively inactive.

Given the significant differences observed between patients with iron deficiency and those with iron sufficiency, we conducted a detailed examination of MAIT cells. Utilizing t-distributed stochastic neighbor embedding (t-SNE) for dimensionality reduction, we investigated the immunophenotype of 25,610 MAIT cells, revealing distinct clusters between iron-sufficient and iron-deficient MAIT cells (Figure 5A). We then identified five clusters that showed apparent differences between the two groups (Figure 5B). Clusters 1 and 5 were more prevalent in iron-deficient patients, whereas clusters 2, 3, and 4 were more common in iron-sufficient patients. The immunophenotypes of these clusters were further analyzed in Figure 5C.

MAIT cells in cluster 1 displayed characteristics more typical of MAIT cells, with higher expression of MAIT cell markers CD161 and Vα7.2. Notably, MAIT cells from iron-deficient patients in this cluster produced more granzyme B than iron-sufficient patients. Similarly, cluster 5 exhibited heightened functional activation, characterized by increased granzyme B, perforin, and IFN-γ production. In particular, MAIT cells from iron-deficient patients produced even higher levels of these molecules. Despite their similarities, clusters 1 and 5 differed in that cluster 5 showed lower expression of CD161.

Clusters 2, 3, and 4 were more abundant in iron-sufficient patients. Like cluster 1, cluster 2 expressed higher levels of CD161 and Vα7.2, but MAIT cells from iron-sufficient patients produced more perforin. Cluster 3 was relatively “silent”, producing similar amounts of IFN-γ and granzyme B and less perforin than other MAIT cells. MAIT cells in cluster 4 were also “silent” but distinguished themselves by expressing lower levels of CD8.

In summary, the active MAIT cell clusters varied significantly between patients with iron deficiency and those with iron sufficiency. Clusters 1 and 5, characterized by higher perforin production, IFN-γ, and granzyme B, were more prevalent in iron-deficient patients. Conversely, cluster 2 exhibited notable cytokine production and was more prevalent in iron-sufficient patients. Additionally, the relatively less active clusters 3 and 4 were more frequently observed in iron-sufficient patients. This suggests that MAIT cells in iron-deficient patients, who may have more severe *M.tb* infections, exhibit a more active state.

## Discussion

In this study, we enrolled a group of pulmonary TB patients in eastern China and observed more severity in iron-deficient patients whose MAIT, Vδ2^+^, and Treg cells showed lower frequency in the periphery while IL-1β and IL-7 were higher. Moreover, serum iron played a protective role during the six-month antituberculosis treatment. Our findings indicated that iron was involved in TB pathogenesis, and iron supplements might be beneficial during the treatment.

MAIT was an unconventional T cell that was discovered 30 years ago. Unlike classic T cells, which recognize antigens by T-cell receptor (TCR) with the help of MHC molecules on the antigen-presenting cells (APC), MAIT is MHC independent, and the evolutionarily conserved MHC class I related molecule (MR1) is used instead during the process.^17^ Antigen recognition by MAIT cells is assisted with more conserved TCR whose α chain consists of TRAV1-2 (Vα7.2) with either TRAJ33/12/20 paired with variable β chains, generally TRBV6-1, TRVB 6-4, or TRBV20.^18^ Besides, most peripheral classic T cells are either CD4 or CD8 positive, and MAIT was regarded as CD8 positive only in the early years, while they were found to be both CD4 or CD8 positive or negative later.^19^

MAIT cells are rare in the periphery, ranging from 1%–10%.^20^ The current study found that MAIT cells occupied only 0.67% of PBMC in iron-sufficient patients, while they were even lower—0.38% in iron-deficient patients. One previous study showed that MAIT cells were numerically depleted in peripheral blood compared with healthy donors.^21^ Where these cells go or are exhausted in TB infection is of great interest. Another study on the rhesus macaque model found that the MAIT cell population expanded and migrated to the site of infection, instigating a Th1 effector response.^22^ However, studies on humans are absent. Whether MAIT cells migrate to the lung and play essential roles remains unclear.

Most MAIT cells are located in the liver (20–50%) and intestine (2–10%), which may be affected by antituberculosis drugs and hepatotoxicity. An essential role of MAIT cells is the homeostasis maintenance of intestinal microecology. MAIT recognizes monomorphic MR1, which presents precursors from the riboflavin (RF) pathway in many bacterial species.^23^ *M.tb* also generates riboflavin metabolites, activating MAIT, resulting in cytokines like IL-17 and IFN-γ release. Based on these findings, MAIT cells can be involved in TB pathogenesis.

A study showed that MAIT cells upregulate CD71 to acquire more iron from the environment upon activation. When iron supply is limited, their proliferation is blocked, metabolic state changes, and cytokine production decreases.^24^ This is consistent with our finding that the percentage of MAIT cells significantly decreased in iron-deficient patients. Research on the impact of iron on Treg cells is more extensive. Tumor-associated Treg cells can expand through iron capture via CD71, and iron deficiency results in decreased CD71 expression, thereby inhibiting Treg cell proliferation.^25^ Microbe-assisted iron uptake promotes Treg cell differentiation in the intestine.^26^ Despite existing studies, the impact and mechanisms of iron on unconventional T cells, such as MAIT and γδ T cells, remain largely unexplored.

IL-1β is essential for immune protection against TB in the early stage of infection, while prolonged production is associated with neutrophil infiltration,^27^ and high IL-1β-expressing genotype is correlated with disease progression and poor treatment outcomes of TB patients.^28^ IL-7 is critical for human T cell lymphopoiesis and enhances the survival of memory T cells.^29^ Higher IL-1β and IL-7 in iron-deficient patients might be involved in neutrophil infiltration and the compensation for decreased unconventional T cells in the periphery, calling for in-depth studies.

Iron deficiency is associated with a higher HRCT score, indicating that iron supplements may be beneficial during the treatment. However, several studies on iron supplements on TB have controversial conclusions.^30^ Excessive iron promotes bacterial growth inside cells.^31^ In animal models, iron supplementation exerted benefits concerning systemic and lung inflammation and markers of anemia of infection,^32^ while another study found that iron supplement decreases proinflammatory cytokine levels and neutrophil recruitment diminishes bacterial load.^31^ In clinical studies, controversial conclusions did exist. A study involving 117 male TB patients with 2 months of 75 mg of elemental iron showed no significance on radiological and clinical improvements in all three groups.^33^ Another study found that iron deficiency without anemia was associated with a 2.89-fold increase in the risk of death.^34^ It is believed that iron has either beneficial or detrimental roles in mycobacteria infection, as it is involved in mycobacterial virulence and the immune cell response of the host. Herein, there is an “iron benefit window” for iron supplements, while at high concentrations, iron can benefit mycobacteria growth and virulence.^35^

### Limitations of the study

One limitation of our study is the subjectivity inherent in the CT scoring system utilized to assess disease progression despite efforts to maintain objectivity. Two radiologists performed this scoring, and while they aimed for consensus, subjective bias remains unavoidable. Future research could benefit from applying artificial intelligence (AI) techniques for image analysis to mitigate this issue, enhancing the consistency and accuracy of assessments.

The study also focused on the peripheral frequency of specific T cell types and cytokine levels in iron deficiency versus iron sufficiency. Future studies should explore the specific mechanisms by which iron influences these cell populations and cytokine profiles better to understand its impact on TB pathogenesis and treatment outcomes.

## Resource availability

### Lead contact

Further information and requests for resources and reagents should be directed to and will be fulfilled by the lead contact Jianming Wang (jmwang@njmu.edu.cn).

### Materials availability

This study did not generate new unique reagents.

### Data and code availability

• This paper does not report the original code.
• Raw data have been deposited at Mendeley Data and are publicly available as of the date of publication. Accession numbers are listed in the key resources table.
• Any additional information required to reanalyze the data reported in this paper is available from the lead contact upon request.

## Acknowledgments

This study was funded by the 10.13039/501100001809National Natural Science Foundation of China (82473693, 81973103), Emergency Special Project of Jiangsu Provincial Department of Science and Technology (BE2023603), Medical Research Project of Jiangsu Health Commission (ZDB2020013), Changzhou Medical Center Grant (CMCM202211).

## Author contributions

Conceptualization, Z.W.; methodology, Z.G. and Z.W.; software, Z.G. and X.S.; investigation, Z.W., Z.G., Q.Z., C.Y., and Q.W.; resources, Y.X. and Z.Z.; writing - original draft, Z.W.; writing - review and editing, J.W.; supervision, J.W. and Z.Z.; funding acquisition, J.W.

## Declaration of interests

All authors declared no competing interests.

## STAR★Methods

### Key resources table

**Table:** 

REAGENT or RESOURCE	SOURCE	IDENTIFIER
**Antibodies**		

Anti-Human CD3-BUV563 (UCHT1)	BD	Cat# 748569; RRID: AB_2872978
Anti-Human γδTCR-APC/Fire 750 (B1)	BioLegend	Cat# 331228; RRID: AB_2650627
Anti-Human Vδ2-BV605 (B6)	BioLegend	Cat# 331430; RRID: AB_2783213
Anti-Human Vα7.2-BV711 (3C10)	BioLegend	Cat# 351732; RRID: AB_2629680
Anti-Human CD161-PE/Cyanine7 (HP-3G10)	BioLegend	Cat# 339918; RRID: AB_11126745
Anti-Human CD4-Spark UV 387 (SK3)	BioLegend	Cat# 344686; RRID: AB_2922562
Anti-Human CD8-BV421 (SK1)	BioLegend	Cat# 344748; RRID: AB_2629584
Anti-Human CD45RA-PerCP/Cyanine5.5 (HI100)	BioLegend	Cat# 304122; RRID: AB_893357
Anti-Human CD197-KIRAVIA Blue 520 (G043H7)	BioLegend	Cat# 353260; RRID: AB_2832694
Anti-Human CD25-PE/Dazzle 594 (BC96)	BioLegend	Cat# 302646; RRID: AB_2734260
Anti-Human CD127-APC (A019D5)	BioLegend	Cat# 351316; RRID: AB_10900804
Anti-Human CD279-BV421 (EH12.2H7)	BioLegend	Cat# 329920; RRID: AB_10960742
Anti-Human CD185-BV785 (J252D4)	BioLegend	Cat# 356936; RRID: AB_2629528
Anti-Human CD196-BV650 (G034E3)	BioLegend	Cat# 353426; RRID: AB_2563869
Anti-Human CD183-PE (G025H7)	BioLegend	Cat# 353706; RRID: AB_10962912
Anti-Human CD194-BV661 (1G1)	BD	Cat# 752522; RRID: AB_2917512
Anti-Human/mouse Granzyme B-APC (QA16A02)	BioLegend	Cat# 372204; RRID: AB_2687028
Anti-Human Perforin-PE (dG9)	BioLegend	Cat# 308106; RRID: AB_314704
Anti-Human IFN-γ-BV650 (4S.B3)	BioLegend	Cat# 502538; RRID: AB_2563608

**Biological samples**		

PBMC and serum from TB patients	NA	NA

**Chemicals, peptides, and recombinant proteins**		

PBMC isolation reagent	Dakewe	Cat# 7111012
CELLSAVING	NCM biotech	Cat# C40100
X-VIVO 15 medium	Lonza	Cat# 04-418Q
Zombie Aqua	Biolegend	Cat# 423101
Human TruStain FcX	Biolegend	Cat# 422302
Brilliant Stain Buffer	BD	Cat# 563794
Fixation Buffer	Biolegend	Cat# 420801
Intracellular Staining Perm Wash Buffer	Biolegend	Cat# 421002
ESAT6-peptide-1:MTEQQWNFAGIEAAA	Sangon	NA
ESAT6-peptide-2:QWNFAGIEAAASAIQ	Sangon	NA
ESAT6-peptide-3:AGIEAAASAIQGNVT	Sangon	NA
ESAT6-peptide-4:AAASAIQGNVTSIHS	Sangon	NA
ESAT6-peptide-5:AIQGNVTSIHSLLDE	Sangon	NA
ESAT6-peptide-6:NVTSIHSLLDEGKQS	Sangon	NA
ESAT6-peptide-7:IHSLLDEGKQSLTKL	Sangon	NA
ESAT6-peptide-8:LDEGKQSLTKLAAAW	Sangon	NA
ESAT6-peptide-9:KQSLTKLAAAWGGSG	Sangon	NA
ESAT6-peptide-10:TKLAAAWGGSGSEAY	Sangon	NA
ESAT6-peptide-11:AAWGGSGSEAYQGVQ	Sangon	NA
ESAT6-peptide-12:GSGSEAYQGVQQKWD	Sangon	NA
ESAT6-peptide-13:EAYQGVQQKWDATAT	Sangon	NA
ESAT6-peptide-14:GVQQKWDATATELNN	Sangon	NA
ESAT6-peptide-15:KWDATATELNNALQ	Sangon	NA
CFP10-peptide-1:MAEMKTDAATLAQEA	Sangon	NA
CFP10-peptide-2:KTDAATLAQEAGNFE	Sangon	NA
CFP10-peptide-3:ATLAQEAGNFERISG	Sangon	NA
CFP10-peptide-4:QEAGNFERISGDLKT	Sangon	NA
CFP10-peptide-5:NFERISGDLKTQIDQ	Sangon	NA
CFP10-peptide-6:ISGDLKTQIDQVEST	Sangon	NA
CFP10-peptide-7:LKTQIDQVESTAGSL	Sangon	NA
CFP10-peptide-8:IDQVESTAGSLQGQW	Sangon	NA
CFP10-peptide-9:ESTAGSLQGQWRGAA	Sangon	NA
CFP10-peptide-10:GSLQGQWRGAAGTAA	Sangon	NA
CFP10-peptide-11:GQWRGAAGTAAQAAV	Sangon	NA
CFP10-peptide-12:GAAGTAAQAAVVRFQ	Sangon	NA
CFP10-peptide-13:TAAQAAVVRFQEAAN	Sangon	NA
CFP10-peptide-14:AAVVRFQEAANKQKQ	Sangon	NA
CFP10-peptide-15:RFQEAANKQKQELDE	Sangon	NA
CFP10-peptide-16:AANKQKQELDEISTN	Sangon	NA
CFP10-peptide-17:QKQELDEISTNIRQA	Sangon	NA
CFP10-peptide-18:LDEISTNIRQAGVQY	Sangon	NA
CFP10-peptide-19:STNIRQAGVQYSRAD	Sangon	NA
CFP10-peptide-20:RQAGVQYSRADEEQQ	Sangon	NA
CFP10-peptide-21:VQYSRADEEQQQALS	Sangon	NA
CFP10-peptide-22:RADEEQQQALSSQMGF	Sangon	NA

**Critical commercial assays**		

LEGENDplex kit Capture Bead A3	Biolegend	Cat# 741096
LEGENDplex kit Capture Bead A4	Biolegend	Cat# 741114
LEGENDplex kit Capture Bead A5	Biolegend	Cat# 741115
LEGENDplex kit Capture Bead A8	Biolegend	Cat# 741100
LEGENDplex kit Capture Bead A10	Biolegend	Cat# 741101
LEGENDplex kit Capture Bead B2	Biolegend	Cat# 741102
LEGENDplex kit Capture Bead B5	Biolegend	Cat# 741105
LEGENDplex kit Capture Bead B9	Biolegend	Cat# 741108
LEGENDplex kit Standard	Biolegend	Cat# 741095
LEGENDplex kit Detection Abs	Biolegend	Cat# 741094
LEGENDplex kits Set A2	Biolegend	Cat# 741110
Human IP-10 ELISA kit	Elabscience	Cat# E-EL-H0050
Human TGF-β1 ELISA kit	Elabscience	Cat# E-EL-0162
Human IL-13 ELISA kit	Elabscience	Cat# E-EL-H0104
Human IL-17 ELISA kit	Elabscience	Cat# E-EL-H5812
Human Ferritin ELISA kit	Elabscience	Cat# E-EL-H0168
Human Hepcidin ELISA kit	Elabscience	Cat# E-EL-H6202

**Deposited data**		

Raw data	This paper	https://data.mendeley.com/preview/spxp2r24cj?a=2953d6f6-ca26-4ed3-a7ec-d8c9a4b9dfe8

**Software and algorithms**		

GraphPad Prism 9.0.2.161	GraphPad	https://www.graphpad.com/
FlowJo 10.8.1	BD	https://www.flowjo.com/
R version 4.3.1	R	https://cran.r-project.org/bin/windows/base/
Origin 2021	OriginLab Corporation	https://originhub.com/#/
Generic Diagramming Platform^36^	BioGDP	https://biogdp.com/

### Experimental model and study participant details

#### TB patients and their PBMC cells

TB patients were diagnosed according to the "People’s Republic of China Health Industry Standard—Diagnosis of Pulmonary Tuberculosis" (WS 288–2017). We collected standardized medical checklists from 808 TB patients, comprising 580 males with a median age of 55 and 228 females with a median age of 49. The severity of TB was assessed, categorizing 666 patients as mild and 142 as severe.

CT scans were obtained from 89 patients (55 males with a median age of 47.5 and 34 females with a median age of 46.5). Plasma samples were collected from 160 patients (122 males with a median age of 55 and 38 females with a median age of 46) to measure plasma ferritin and hepcidin levels; among these, 117 cases were classified as mild and 43 as severe.

Additionally, PBMCs were collected from 48 patients (30 males and 18 females, all with a median age of 51) to investigate differences in T cell subsets between iron-deficient (*n* = 23) and iron-sufficient (*n* = 25) patients. All participants are Chinese.

Age and sex did not influence the outcomes, as these factors were well-matched between groups, and no statistically significant differences were observed.

Sample collection was conducted in accordance with the Declaration of Helsinki and was approved by the Ethics Committee of Changzhou Third People’s Hospital (approval number: 02A-A20230002) and the Ethics Committee of Nanjing Medical University (approval number: 2024-014). Informed consent was obtained from all participants.

### Method details

#### Study design and participants

We recruited 808 pulmonary TB patients from Changzhou Third People’s Hospital during 2018–2022. We categorized patients into two groups (mild and severe) based on disease severity and analyzed the relation between disease severity and serum iron level using a case-control study design. A subset of 160 patients was enrolled to assess the relationships between serum iron, ferritin, and hepcidin. We followed 89 patients to record CT images at the time of diagnosis and 2 months and 6 months after the treatment using a cohort study design. We further enrolled 48 patients to isolate PBMCs for immunity analyses. Patients with cancer, immunodeficiency, or pregnancy were excluded. This study was permitted by the Ethics Committee of Changzhou Third People’s Hospital (02A-A20230002) and the Ethics Committee of Nanjing Medical University (2024-014).

#### TB severity assessment

Bandim TB score is a widely used scoring system to evaluate TB severity briefly.^37^ However, mid-upper arm circumference (MUAC) within the score was inaccessible to patients in our hospital. Therefore, a modified Bandim TB score, which evaluates TB severity without MUAC, was employed instead. The modified Bandim TB score consisted of five symptoms and five clinical signs: cough, hemoptysis, chest pain, dyspnea, night sweats and body temperature >37°C, pulse >90/min, auscultation abnormity, BMI＜18 kg/m^2^, BMI＜16 kg/m^2^, anemia.^38^ Any positive findings above got one point, and the TB score was the sum of these points. TB severity was divided into mild (score <5) and severe (score ≥5).

#### High-resolution CT (HRCT) score

HRCT score considers 7 different pulmonary lesions observed on CT: consolidation, micro nodule, nodule, ground-glass opacity (GGO), cavity, bronchiectasis, and parenchymal band.^39^ Each lesion was estimated with a score from 0 to 4 according to the area. The whole lung was divided into upper, middle, and lower parts for each half. The HRCT score was the sum of 7 lesions on 6 parts, namely, from 0 to 168. The assessment was done by two radiologists (Dr. Wei, 3 years of work experience; Dr. Ding, 2 years of work experience). Disagreements were solved by consultation with the chief physician of the radiology department.

#### PBMC isolation and stimulation

We collected 5 mL of whole blood from each patient before treatment with EDTA tubes and mixed it with 5 mL PBS. The mixture was then added onto 5 mL of separation reagent, followed by centrifugation at 800 *g* for 30 min. The PBMC aggregated middle layer was aspirated into a new tube and washed twice with PBS. The cells were finally collected and cryopreserved in liquid nitrogen until use. The isolation procedure was done within 6 h after blood sampling, and PBMC was kept within 3 months before use.

PBMC was thawed in a 37°C water bath and washed twice with 10 mL prewarmed X-VIVO 15 medium. After cell counting, PBMC was cultured in an ultra-low-attachment 24-well plate with 1 mL X-VIVO 15 media for 12 h. TB antigen CFP-10 and ESAT-6 peptides (synthesized by Sangon Biotech) were added at 2 μg/10^6^ cells and cultured at 37°C, 5% CO_2_ for 12 h.

#### Flow cytometry assay

PBMC was divided into two stain panels for CD4^+^ T cells (panel 1) and CD8^+^ T cells (panel 2). The cultured PBMC was centrifuged at 500 g for 5 min, washed with PBS, and resuspended in 200 μL PBS flowed by Zombie Aqua stain for 20 min to distinguish the live and dead cells. The cells were later blocked with Human TruStain FcX for 10 min. Antibodies were diluted in Brilliant Stain Buffer according to the manufacturer’s instructions and stained for 20 min in the dark before flow cytometer analyses. For panel 1, the cell markers stained were CD3, Vδ2, γδTCR, CD161, Vα7.2, CD4, CD45RA, CCR7, CD25, CD127, PD-1, CXCR5, CCR6, CXCR3, CCR4. For panel 2, these markers were CD3, Vδ2, γδTCR, CD161, Vα7.2, CD4, CD8, CD45RA, CCR7, Granzyme B, Perforin, and IFN-γ while the last three were further stained after permeation and fixation by Fixation Buffer and Intracellular Staining Perm Wash Buffer. Cell gating was performed using FlowJo, and indistinct clusters were identified with fluorescence minus one (FMO) controls. Dimensionality reduction analysis was conducted using t-Distributed Stochastic Neighbor Embedding (t-SNE) functions within FlowJo by concatenating all 25,610 MAIT cells from all patients.

#### Cytokine and ferritin, hepcidin measurement

PBMC culture supernate, which was stimulated by TB antigen peptides, was used for cytokine detection. IL-1β, IL-2, IL-6, IL-7, IL-10, GM-CSF, IFN-γ, TNF-α were measure by BioLegend LEGENDplex kits. Briefly, the standard substance was diluted with assay buffer, and 25 μL beads, 25 μL assay buffer, and 25 μL sample or standard substance were added and mixed in each well. The plate was incubated in a shaker at room temperature for 2 h. Twenty-five microliter detection antibodies were added after washing with wash buffer and incubated for 1 h. Twenty-five microliters of SA-PE were then added and incubated for 30 min. After washing, the plate was loaded on the flow cytometer. IP-10, TGF-β1, IL-13, and IL-17 were measured by ELISA kits according to manufacturer’s instructions. The concentrations measured were finally adjusted according to PBMC numbers.

Serum, collected from PBMC isolation and diluted three times, was used for ferritin and hepcidin measurements using ELISA kits according to the manufacturer’s instructions.

### Quantification and statistical analysis

Serum iron and the checklists of all patients were output from the case system of Changzhou Third People’s Hospital. The normality of data was first tested by the Shapiro-Wilk test and shown as mean (SD) for normal ones, mean (IQR) for skewed ones, and n(%) for categorical variables. The differences between groups were analyzed using the Wilcoxon test for continuous variables and Fisher’s exact test for dichotomous variables. The relationship between serum iron and HRCT score was evaluated by generalized estimating equations (GEE), where the serum iron level was used as an independent variable, HRCT scores as the dependent variable, and sex or age as a covariate. Data analyses were performed in R version 4.3.1, and the test level was set at 0.05. For figures ∗, *p* < 0.05, ∗∗, *p* < 0.01, ∗∗∗, *p* < 0.001, ns, not significant.
