# Supplementary material for: Relationship between iron deficiency and severity of tuberculosis: Influence on T cell subsets
Source: iScience. 2024 Dec 30;28(2):111709. doi: 10.1016/j.isci.2024.111709 (PMC11783395; doi:10.1016/j.isci.2024.111709)
Supplement: Document S2. Tables S1 and S2 [file mmc1.pdf]

iScience, Volume *28*

Supplemental information

Relationship between iron deficiency and severity of tuberculosis: Influence on T cell subsets

Zheyue Wang, Zhenpeng Guo, Qiang Zhang, Chenchen Yang, Xinling Shi, Qin Wen, Yuan Xue, Zhixin Zhang, and Jianming Wang

Table S1: Detailed characteristics of mild and severe TB, related to Table 1

Characteristics	Mild TB (N=666)	Severe TB (N=142)	Normal range	*P* value
**Demography**				
sex			-	0.078
male	469 (70.4%)	111 (78.2%)	-	
female	197 (29.6%)	31 (21.8%)	-	
age	53.5 [34.0, 68.0]	57.0 [32.5, 73.0]	-	0.148
**Comorbidities**				
hypertension	146 (21.9%)	21 (14.8%)	-	0.073
diabetes	120 (18.0%)	20 (14.1%)	-	0.316
**Liver function**				
ADA (U/L)	10.6 [8.4, 14.7]	14.9 [10.7, 19.0]	[2, 18]	**<0.001**
AFU (U/L)	23.4 [19.4, 27.8]	20.0 [16.0, 25.3]	[0, 40]	**<0.001**
AKP (U/L)	75.0 [62.0, 91.0]	80.0 [65.0, 98.0]	[35, 100]	**0.018**
ALT (U/L)	14.6 [10.2, 22.6]	13.9 [9.0, 26.8]	[7, 40]	0.917
AST (U/L)	16.0 [13.0, 20.0]	18.0 [13.0, 26.0]	[13, 35]	**0.026**
GGT (U/L)	20.6 [14.2, 33.7]	30.6 [19.8, 48.2]	[7, 45]	**<0.001**
LDH (U/L)	157 [138, 180]	169[149, 204]	[109, 245]	**<0.001**
5' nucleotidase (U/L)	1.4 [1.1, 1.7]	1.5 [1.2, 2.3]	[0, 10]	**0.005**
TBIL (μmol/L)	9.50 [6.80, 12.60]	8.60 [6.53, 11.60]	[3.4, 22]	0.078
DBIL (μmol/L)	4.00 [3.20, 5.00]	4.10 [3.40, 5.62]	[1.7, 10.3]	0.111
IBIL (μmol/L)	5.30 [3.42, 7.50]	4.40 [2.92, 6.00]	[1.7, 11.7]	**<0.001**
glycocholic acid (mg/L)	1.30 [0.90, 1.60]	1.30 [0.92, 1.70]	[0, 2.7]	0.856
total bile acid (μmol/L)	3.50 [2.40, 5.77]	3.95 [2.30, 5.97]	[0, 9.67]	0.274
cholinesterase (IU/L)	6784 [5791, 7888]	5170 [4063, 6356]	[3700, 13200]	**<0.001**
**Renal function**				
urea (mmol/L)	4.74 [3.81, 5.88]	4.48 [3.32, 5.54]	[2.9, 8.2]	**0.016**
creatinine (μmol/L)	61.6 [53.2, 71.7]	58.8 [48.3, 69.1]	[35, 80]	**0.027**
cystatin C (mg/L)	0.97 [0.87, 1.12]	1.01 [0.89, 1.16]	[0.55, 1.05]	**0.046**
uric acid (μmol/L)	290 [238, 357]	247 [191, 325]	[210, 420]	**<0.001**
β2-microglobulin (mg/L)	2.00 [1.51, 2.50]	2.60 [2.00, 3.40]	[1, 3]	**<0.001**
**Cardiac function**				
BNP (pg/ml)	32.2 [17.3, 61.4]	38.3 [21.9, 84.6]	[0, 100]	**0.023**
**Nutritional status**				
glucose (mmol/L)	4.90 [4.40, 5.60]	5.00 [4.52, 5.76]	[3.89, 6.11]	0.144
total protein (g/L)	66.7 [63.2, 70.6]	65.8 [60.5, 69.8]	[65, 85]	**0.03**
albumin (g/L)	**37.4 [33.6, 40.3]**	**31.4 [27.8, 34.7]**	**[40, 55]**	**<0.001**
globulin (g/L)	29.6 [26.1, 32.8]	33.7 [29.6, 38.5]	[20, 40]	**<0.001**
A/G	1.3 [1.0, 1.5]	**0.9 [0.8, 1.1]**	**[1.2, 2.4]**	**<0.001**
prealbumin (mg/dL)	**19.6 [14.9, 23.9]**	**12.1 [8.9, 17.5]**	**[20, 40]**	**<0.001**
phosphorus (mmol/L)	1.12 [0.97, 1.24]	1.03 [0.92, 1.16]	[0.8, 1.45]	**<0.001**
calcium (mmol/L)	2.17 [2.10, 2.25]	2.09 [2.01, 2.20]	[2.0, 2.5]	**<0.001**
potassium (mmol/L)	3.91 [3.69, 4.15]	3.90 [3.63, 4.22]	[3.5, 5.3]	0.818
sodium (mmol/L)	141 [139, 142]	138 [135, 141]	[137, 147]	**<0.001**
chlorine (mmol/L)	105 [103, 107]	102 [98, 104]	[99, 110]	**<0.001**
magnesium (mmol/L)	0.88 [0.81, 0.93]	0.84 [0.78, 0.90]	[0.62, 1.05]	**<0.001**
iron (μmol/L)	**10.20 [6.58;15.10]**	**5.04 [3.58, 8.05]**	**female [9, 27]**<br>**male [11, 30]**	**<0.001**
transferrin (g/L)	2.00 [1.70, 2.20]	**1.60 [1.30, 1.87]**	**[2, 4]**	**<0.001**

CO_2 (mmol/L)	25.2 [22.7, 27.0]	25.9 [23.1, 28.1]	[20, 29]	0.059
**Serum lipids**				
total cholesterol (mmol/L)	3.86 [3.42, 4.50]	3.44 [3.02, 4.00]	[3.4, 5.8]	**<0.001**
triglyceride (mmol/L)	1.04 [0.75, 1.39]	0.96 [0.80, 1.25]	[0.56, 1.7]	0.368
HDL (mmol/L)	1.04 [0.86, 1.25]	0.82 [0.69, 1.03]	[0.78, 2]	**<0.001**
LDL (mmol/L)	2.29 [1.92, 2.83]	2.01 [1.70, 2.50]	[0, 3.7]	**0.002**
apolipoprotein A (g/L)	**0.87 [0.76, 1.01]**	**0.68 [0.61, 0.82]**	**[1, 1.6]**	**<0.001**
apolipoprotein B (g/L)	0.70 [0.61, 0.84]	0.69 [0.62, 0.80]	[0.6, 1.2]	0.858
lipoprotein a (mg/L)	141 [70.5, 298]	208 [96.5, 470]	[0, 300]	**0.002**
free fatty acid (mmol/L)	0.37 [0.25, 0.48]	0.44 [0.28, 0.54]	[0.1, 0.77]	**0.014**
**Red blood cells**				
erythrocyte (10^12/L)	4.4 [4.0, 4.8]	4.1 [3.8, 4.5]	[4.1, 5.3]	**<0.001**
platelet (10^9/L)	243 [198, 296]	292 [226, 365]	[150, 407]	**<0.001**
hematocrit HCT (%)	39.5 [35.6, 42.4]	35.3 [32.1, 39.0]	[36, 47]	**<0.001**
MCH (pg)	29.5 [28.5, 30.7]	28.4 [26.9, 29.7]	[25, 34]	**<0.001**
MCHC (g/L)	330.0 [324.0, 337.0]	328.0 [320.0, 334.0]	[310, 355]	**0.001**
MCV (fL)	89.0 [86.5, 92.1]	86.6 [83.1, 90.5]	[80, 100]	**<0.001**
RDW-CV (%)	12.7 [12.2, 13.4]	13.1 [12.4, 14.5]	[11.6, 14.6]	**<0.001**
PDW (%)	**11.9 [10.5, 13.4]**	**10.7 [9.53, 11.9]**	**[14.8, 17.2]**	**<0.001**
MPV (fL)	10.4 [9.70, 11.1]	9.85 [9.30, 10.5]	[7.6, 13.2]	**<0.001**
PCT (%)	0.25 [0.21, 0.30]	0.30 [0.23, 0.36]	[0.108, 0.282]	**<0.001**
**Immunity**				
CRP (mg/L)	**5.3 [0.9, 26.7]**	**51.1 [26.8, 81.8]**	**[0, 5]**	**<0.001**
leukocyte (10^9/L)	5.9 [5.0, 7.3]	6.5 [5.2, 8.3]	[4.1, 11]	**0.006**
lymphocyte (10^9/L)	1.5 [1.1, 1.9]	1.0 [0.7, 1.3]	[1.2, 3.8]	**<0.001**
neutrophil (10^9/L)	3.7 [2.8, 4.8]	4.7 [3.5, 6.0]	[1.8, 8.3]	**<0.001**
monocyte (10^9/L)	0.5 [0.4, 0.7]	0.7 [0.5, 0.8]	[0.14, 0.74]	**<0.001**
eosinophils (10^9/L)	0.11 [0.07, 0.19]	0.07 [0.02, 0.13]	[0, 068]	**<0.001**
basophil (10^9/L)	0.03 [0.02, 0.04]	0.02 [0.02, 0.03]	[0, 0.07]	**0.002**
IgM (g/L)	0.97 [0.70, 1.28]	0.85 [0.64, 1.04]	[0.29, 3.44]	0.205
IgA (g/L)	2.32 [1.80, 3.07]	3.08 [2.16, 3.68]	[0.72, 4.29]	**0.004**
IgG (g/L)	13.4 [11.3, 15.4]	14.1 [11.1, 16.0]	[8, 17]	0.362
complement 3 (g/L)	0.91 [0.76, 1.08]	0.96 [0.80, 1.15]	[0.79, 1.52]	0.331
complement 4 (g/L)	0.24 [0.20, 0.29]	0.27 [0.22, 0.34]	[0.16, 0.38]	0.098

Table S2: HRCT scores of 0, 2 and 6 after the antituberculosis treatment, related to Figure 2 and Table 3

Variables	0-month treatment	2-months treatment	6-months treatment
micro nodule	3 [2, 5]	3 [1, 4]	2 [1, 3]
nodule	0 [0, 2]	0 [0, 2]	0 [0, 1]
consolidation	2 [1, 5]	2 [1, 3]	1 [0, 3]
GGO	0 [0, 0]	0 [0, 0]	0 [0, 0]
cavity	0 [0, 0]	0 [0, 0]	0 [0, 0]
bronchiectasis	0 [0, 2]	0 [0, 2]	0 [0, 2]
parenchymal bands	0 [0, 0]	0 [0, 0]	0 [0, 0]
total HRCT score	9 [4, 14]	7 [4, 10]	4 [2, 8]

Data were shown as mean, interquartile range (IQR).

GGO, ground-glass opacity
